# Trends in female genital mutilation (FGM) among Senegalese women and their daughters : a secondary analysis of Senegal DHS from 2015 to 2023

**DOI:** 10.1186/s12889-025-23975-8

**Published:** 2025-08-19

**Authors:** Ndeye Mareme Sougou, Mouhamadou Faly Ba, Fatou Bintou Diongue, Amadou Ibra Diallo, Ibrahima Ndiaye, Ibrahima Seck

**Affiliations:** 1 Department of Preventive medicine and Public Health, Université Cheikh Anta Diop de Dakar (UCAD), Dakar, Senegal; 2 Institute of health and development, Faculty of medicine, Université Cheikh Anta Diop de Dakar (UCAD), Dakar, Senegal; 3 International Research Laboratory (IRL3189) Environment, Health and Societies (ESS), Cheikh Anta Diop University of Dakar (UCAD), Dakar, Senegal

**Keywords:** Trends, Female genital mutilation, Senegal

## Abstract

**Background:**

Female genital mutilation (FGM) is a deeply rooted practice in Senegal, generally affecting girls at a very young age. The prevalence of FGM has remained virtually unchanged for at least two decades. The aim of this study is to identify the factors associated with the evolution of this practice in Senegal.

**Methods:**

This study is a secondary analysis of data extracted from the Senegalese Demographic and Health Surveys (DHS) from 2015 to 2023. The record individual file of women aged 15 to 49 was used for the analysis. For both women aged 15-49 and their daughters, the dependent variable was "being circumcised". This is a binary qualitative variable which was coded as"Yes" if the woman and/or her daughter had been circumcised.

A descriptive analysis was performed. A multivariate analysis was performed to determine the Adjusted Odds Ratios (ORaj) and estimate the corresponding 95% confidence intervals (CI) for all variables. Adjustment was made on a yearly basis.

**Results:**

The prevalence of female circumcision was 24.2% in 2015, 22.7% in 2016, 24.0% in 2017, 23.3% in 2018, 25.2% in 2019 and 20.1% in 2023. The majority of women who undergo FGM do so between the ages of 0 and 9. The most frequent form of mutilation is "flesh removed from genital area", the frequency of which rises from 58.3% in 2015 to 76.5% of cases of mutilation in 2023. Infibulation (genital area sewn closed), which was the least common form of mutilation at 7% in 2015, will increase to 25.6% in 2023.

The factors associated with the development of FGM among Senegalese women were region of residence and socio-economic level.

**Conclusion:**

Our study has shown that the fact that the mother has been circumcised is a risk factor for the daughter. However, the level of wealth and education of women and their husbands would appear to be protective factors against the development of FGM in girls. To bend the curve, political decision-makers need to take targeted action in hotspot regions, considering aspects linked to women's empowerment.

## Introduction

The UNICEF estimates that more than 230 million girls and women are victims of genital mutilation (FGM) worldwide as of 2024 [1]. Yet female genital mutilation causes severe complications and can even lead to death [2]. Beyond the medical aspect, this practice is also considered a violation of internationally recognised human rights [3]. In the African region, around 35% of girls and women aged between 15 and 49 have undergone FGM, including around 16.7% of girls aged between 0 and 14 between 2012 and 2020 [4]. In Senegal, female genital mutilation (FGM) is a deeply rooted practice that generally affects girls at a very young age. The prevalence of FGM has remained virtually unchanged for at least two decades. Nearly two million girls and women have undergone FGM in Senegal [5]. According to the latest results of the DHS 2019, the prevalence of FGM was 16.1% among girls aged 10–14 and 25.2% among women aged 15–49 [6]. In Senegal, however, many efforts have been made since 1970 to combat FGM. These include awareness-raising programmes and the enactment of the law against FGM which, since 1999, prohibits the practice and sentences excisers to 6 months’ imprisonment for setting an example (Article 299 bis of the Penal Code (law no. 99 − 05 of 29 January 1999)) [7]. Despite these interventions, FGM continues to be practised in Senegal.

Several studies have examined the factors influencing the practice of FGM. The determinants identified were socio-demographic (age, place of residence, education), socio-economic and socio-cultural (marriage, religion**)** factors [8, 9]. It has been shown that women with better financial resources or family wealth were less likely to be circumcised [9]. In addition, being younger and better educated protects against FGM [8, 10]. In addition, cultures that place a high value on preserving virginity, reducing premarital sex and early pregnancy, and minimising the risk of extramarital relations are more likely to encourage FGM [10–12]. However, identifying the factors associated with the development of this practice in Senegal could help refine control strategies and curb the stagnating trend.

The aim of this study is to analyse changes in the practice of female circumcision in Senegal between 2015 and 2023 and to identify the factors associated with these changes.

Nevertheless, significant challenges remain in efforts to reduce FGM prevalence in Senegal. These include deeply rooted cultural beliefs, clandestine practices that have emerged in response to legal restrictions, intergenerational pressure, limited resources for awareness programs, and geographical disparities in intervention coverage. Understanding these challenges is crucial for developing more effective strategies to combat this practice.

## Methodology

This study concerns Senegal. Senegal is located in the extreme west of the African continent. It covers an area of 196,712 km² with a western coastline of over 700 km. It is bordered to the north by the Islamic Republic of Mauritania, to the east by the Republic of Mali, to the south by the Republic of Guinea and the Republic of Guinea Bissau, and to the west by the Atlantic Ocean. The Republic of the Gambia is an enclave 25 km wide and almost 300 km long within Senegalese territory [14].

Senegal is subdivided into 14 regions and the population is structured according to a variety of socio-cultural characteristics, with many ethnic groups (Wolof, Fulbe, Serer, Joola, etc.) [15]. Each of these communities has distinct cultural influences on Senegalese society, which can include differing traditions regarding practices such as FGM.

### Type of study and study period

This study is a secondary analysis of data extracted from the Senegalese Demographic and Health Surveys (DHS) from 2015 to 2023. The record individual file of women aged 15 to 49 was used for the analysis [16].

### Study population

This work focused on women aged between 15 and 49 found in households.

### Sampling

Inclusion and non-inclusion criteria

All women aged 15–49 living in the household visited were included in the study. Non-inclusion criteria were absence or illness during the collection period or refusal to participate.

### Sample size

The sample sizes obtained were respectively in 2015 (*n* = 8851), 2016 (*n* = 8865), 2017 (*n* = 16787), 2018 (*n* = 9414), 2019 (*n* = 8649) and 2023 (16583). This makes a total of 69,149 women included in the study.

### Sampling conception

**Fig. 1:**
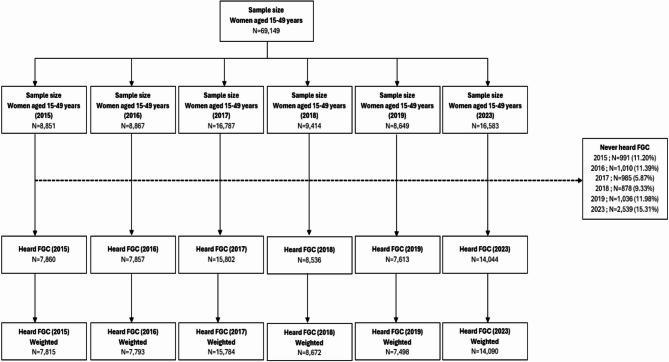
Diagram of study-flow

Each DHS survey employed a stratified two-stage cluster sampling design. Initially, the country was first stratified by urban and rural areas within each of the 14 administrative regions, creating 28 sampling strata. In the first stage and within each stratum, clusters (Primary Survey Units (PSUs)) were drawn from the list of enumeration areas (EAs) established during the 2013 General Census of Population and Housing, Agriculture and Livestock, using a systematic draw with probability proportional to size, the size of the PSUs being the number of households. A count of households in each of these clusters provided a list of households from which a second-stage sample of 22 households per cluster was drawn, in both urban and rural areas, with systematic drawing with equal probability. All women aged 15–49 in the selected households who consent are eligible for individual interviews [16] (Fig. 1).

### Analysis

For women aged 15–49, the dependent variable was “being circumcised”. This is a binary qualitative variable which was coded as “Yes” if the woman had been circumcised. For the respondents’ daughters, the dependent variable “being circumcised” is a qualitative binary variable that was coded as “Yes” if the respondent’s daughter had been circumcised. Information on both the woman’s and the girl’s circumcision was based on the respondent’s declaration.

Regarding the description of the practice of female circumcision, the answer modalities to the question “Who performed the circumcision?” were “Health professionals”, which referred to “doctor” and “nurse and midwife”, and “other health professionals”, which referred to other medical professionals who can provide care.

The independent variables were the mother’s individual characteristics: region of residence, religion. Religion was dichotomised as ‘Muslim’ or ‘other’, and the age of the participants. Age was analysed by 5-year age groups (15–19; 20–24; 25–29; 30–34; 35–39; 40–44; 45–49) and marital status. It also included the mother’s socio-economic characteristics (level of education, occupation, husband’s level of education and wealth quintile. The measure of socio-economic well-being was subdivided into 5 quintiles (very poor, poor, average, rich and very rich). Media exposure was also an independent variable.

Quantitative data were analysed using R software version 4.2.1.

A descriptive analysis was carried out on all the data collected. The qualitative variables were described by their frequencies (absolute and relative). The results were disaggregated by year and all the parameters were obtained considering the weighting and the complex sampling plan. The following key indicators were sought. The chi2 test with the Rao and Scott correction was used to search for associations between the dependent and independent variables with an alpha risk of 5%. Finally, we modelled the dependent variables using a mixed-effects multiple logistic regression model. Clusters were considered as a random effect to account for unexplained variability at the community level. We used this multivariate analysis to determine Adjusted Odds Ratios (ORaj) and estimated the corresponding 95% confidence intervals (CI) for all variables. The adjustment was made on a yearly basis.

## Results

### Socio-economic and demographic characteristics of women

The majority of women were uneducated, representing 50.9% in 2015 to 43.5%. Married women were more numerous. Women of the Muslim faith represented 94.7% in 2015 to 96.8% in 2023. (see Table 1)

**Table 1:** Individual characteristics of women aged 15 to 49 (Senegal, DHS 2015 to 2023)

Characteristics	2015, *N* = 8 851^a^ *n*(%)	2016, *N* = 8 865^a^ *n*(%)	2017, *N* = 16 787^a^ *n*(%)	2018, *N* = 9 414^a^ *n*(%)	2019, *N* = 8 649^a^ *n*(%)	2023, *N* = 16 583^a^ *n*(%)	Total, *N* = 69,149^a^ *n*(%)
**Age in 5-year groups**							
15–19	2 003 (22.6)	1 998 (22.5)	3 728 (22.2)	2 056 (21.8)	1 906 (22.0)	3 877 (23.4)	15 568 (22.5)
20–24	1 641 (18.5)	1 664 (18.8)	3 080 (18.3)	1 686 (17.9)	1 655 (19.1)	3 230 (19.5)	12 954 (18.7)
25–29	1 667 (18.8)	1 524 (17.2)	2 808 (16.7)	1 591 (16.9)	1 383 (16.0)	2 416 (14.6)	11 389 (16.5)
30–34	1 247 (14.1)	1 318 (14.9)	2 590 (15.4)	1 387 (14.7)	1 290 (14.9)	2 216 (13.4)	10 048 (14.5)
35–39	1 030 (11.6)	998 (11.3)	1 878 (11.2)	1 153 (12.3)	1 081 (12.5)	2 143 (12.9)	8 283 (12.0)
40–44	772 (8.7)	806 (9.1)	1 586 (9.4)	868 (9.2)	725 (8.4)	1 598 (9.6)	6 354 (9.2)
45–49	492 (5.6)	557 (6.3)	1 117 (6.7)	673 (7.2)	609 (7.0)	1 104 (6.7)	4 552 (6.6)
**Highest educational level**							
No education	4 505 (50.9)	4 310 (48.6)	7 749 (46.2)	4 304 (45.7)	4 090 (47.3)	7 220 (43.5)	32 178 (46.5)
Primary	1 927 (21.8)	1 967 (22.2)	3 861 (23.0)	2 070 (22.0)	1 824 (21.1)	3 416 (20.6)	15 065 (21.8)
Secondary	2 136 (24.1)	2 241 (25.3)	4 431 (26.4)	2 554 (27.1)	2 364 (27.3)	5 070 (30.6)	18 794 (27.2)
Higher	283 (3.2)	347 (3.9)	745 (4.4)	486 (5.2)	371 (4.3)	877 (5.3)	3 110 (4.5)
**Husband/partner’s education** **level**							
No education	4 718 (76.8)	4 822 (76.4)	7 579 (69.6)	4 156 (69.1)	4 054 (71.6)	7 559 (70.8)	32 889 (72.0)
Primary	648 (10.6)	607 (9.6)	1 484 (13.6)	776 (12.9)	640 (11.3)	1 260 (11.8)	5 414 (11.8)
Secondary	536 (8.7)	584 (9.3)	1 252 (11.5)	738 (12.3)	635 (11.2)	1 150 (10.8)	4 896 (10.7)
Higher	240 (3.9)	297 (4.7)	580 (5.3)	343 (5.7)	330 (5.8)	701 (6.6)	2 491 (5.5)
**Respondent’s occupation (grouped**)							
Not working	3 957 (44.7)	4 253 (48.0)	7 012 (41.8)	3 928 (41.7)	3 995 (46.2)	9 580 (57.8)	32 725 (47.3)
Working	4 894 (55.3)	4 612 (52.0)	9 775 (58.2)	5 486 (58.3)	4 654 (53.8)	7 003 (42.2)	36 424 (52.7)
**Wealth index**							
Poorest	1 509 (17.0)	1 475 (16.6)	2 768 (16.5)	1 508 (16.0)	1 415 (16.4)	2 703 (16.3)	11 378 (16.5)
Poorer	1 579 (17.8)	1 603 (18.1)	2 984 (17.8)	1 659 (17.6)	1 529 (17.7)	2 983 (18.0)	12 337 (17.8)
Middle	1 757 (19.8)	1 749 (19.7)	3 310 (19.7)	1 908 (20.3)	1 684 (19.5)	3 362 (20.3)	13 771 (19.9)
Richer	1 886 (21.3)	1 991 (22.5)	3 581 (21.3)	2 065 (21.9)	1 870 (21.6)	3 552 (21.4)	14 946 (21.6)
Richest	2 121 (24.0)	2 046 (23.1)	4 144 (24.7)	2 273 (24.1)	2 151 (24.9)	3 983 (24.0)	16 718 (24.2)
**Current marital status**							
Not married	3 140 (35.5)	3 047 (34.4)	5 910 (35.2)	3 421 (36.3)	2 998 (34.7)	5 986 (36.1)	24 502 (35.4)
Married	5 711 (64.5)	5 818 (65.6)	10 877 (64.8)	5 993 (63.7)	5 651 (65.3)	10 597 (63.9)	44 647 (64.6)
**Religion**							
Muslim	8 380 (94.7)	8 530 (96.2)	16 160 (96.3)	9 053 (96.2)	8 409 (97.2)	16 058 (96.8)	66 590 (96.3)
Other	471 (5.3)	335 (3.8)	627 (3.7)	361 (3.8)	240 (2.8)	525 (3.2)	2 559 (3.7)

^a^ n (%)

### FGM practice in Senegal from 2015 to 2023

In Africa, in general, the prevalence of FGM has decreased but remains persistent. The prevalence of FGM was 24.2% in 2015, 22.7% in 2016, 24.0% in 2017, 23.3% in 2018, 25.2% in 2019 and 20.1% in 2023. The majority of women who undergo FGM do so between the ages of 0 and 9. (See Table 2)

Prevalence among girls of circumcised women varies between 2015 (7.8%), 2016 (7.1%), 2017 (6.8%), 2018 (6.4%), 2019 (7.8%) and 2023 (6%). See Table 2.

The most frequent form of mutilation is “flesh removed from genital area”, the frequency of which will rise from 58.3% in 2015 to 76.5% of cases of mutilation in 2023. Infibulation (genital area sewn closed), which was the least common form of mutilation at 7% in 2015, will increase to 25.6% in 2023. (See Table 2)

Most excisers are traditional circumcisers. In 2023, however, a new category of circumcisers emerged: health professionals (58, or 1.7%). (See Table 2)

**Table 2:** Prevalence of female genital mutilation (Senegal, DHS 2015 to 2023)

characteristics	2015, *N* = 8 851	2016, *N* = 8 865	2017, *N* = 16 787	2018, *N* = 9 414	2019, *N* = 8 649	2023, *N* = 16 583
**Respondent circumcised**						
Yes	2 146 (24,2%)	2 013 (22,7%)	4 030 (24,0%)	2 196 (23,3%)	2 181 (25,2%)	3 330 (20,1%)
**Type of FGM**						
Flesh removed from genital area	1 251 (58,3%)	1 150 (57,1%)	2 482 (61,6%)	1 338 (61,0%)	1 301 (59,7%)	2 548 (76,5%)
Genital area just nicked without removing any flesh	259 (28,9%)	321 (37,1%)	536 (34,7%)	252 (29,4%)	280 (31,8%)	124 (15,9%)
Genital area sewn closed	149 (7,0%)	143 (7,1%)	415 (10,3%)	151 (6,9%)	198 (9,1%)	853 (25,6%)
**Age at circumcision (in years)**						
0–4	291 (13,6%)	283 (14,1%)	446 (11,1%)	216 (9,9%)	239 (11,0%)	524 (15,7%)
05–09	370 (17,2%)	263 (13,1%)	488 (12,1%)	211 (9,6%)	228 (10,4%)	420 (12,6%)
10–14	141 (6,6%)	127 (6,3%)	174 (4,3%)	84 (3,8%)	58 (2,7%)	116 (3,5%)
15–19	15 (0,7%)	19 (0,9%)	23 (0,6%)	8 (0,4%)	6 (0,3%)	6 (0,2%)
20–24	1 (0,0%)	7 (0,3%)	6 (0,1%)	1 (0,0%)	2 (0,1%)	0 (0,0%)
25–29	2 (0,1%)	0 (0,0%)	1 (0,0%)	0 (0,0%)	0 (0,0%)	0 (0,0%)
During infancy (don’t remember age)	1 159 (54,0%)	1 170 (58,1%)	2 763 (68,6%)	1 598 (72,8%)	1 611 (73,9%)	1 706 (51,2%)
**Person who performed circumcision**						
Health professional	0 (0,0%)	0 (0,0%)	0 (0,0%)	0 (0,0%)	0 (0,0%)	0 (0,0%)
Other health professional	0 (0,0%)	0 (0,0%)	0 (0,0%)	0 (0,0%)	0 (0,0%)	58 (1,7%)
Traditional “circumciser	2 015 (93,9%)	1 867 (92,8%)	3 835 (95,2%)	2 105 (95,9%)	2 102 (96,4%)	3 125 (93,8%)
Traditional birth attendant	8 (0,4%)	7 (0,3%)	24 (0,6%)	4 (0,2%)	0 (0,0%)	0 (0,0%)
Other traditional	123 (5,8%)	138 (6,9%)	171 (4,2%)	87 (3,9%)	79 (3,6%)	147 (4,4%)
**Daughters circumcised**						
Yes	693 (7,8%)	630 (7,1%)	1 142 (6,8%)	604 (6,4%)	672 (7,8%)	987 (6,0%)

### Factors associated with FGM from 2015 to 2023

#### Women aged 15–49 in senegal, 2015–2023

The factors associated with the development of FGM among Senegalese women were region of residence and socio-economic level. Thus, living in one region or another may be a protective factor or a risk factor. Living in a central region is a protective factor against the occurrence of FGM. These are the regions of Thiès (Oraj = 0.21 [0.15–0.28]), Kaolack (Oraj = 0.3 [0.21–0.42]), Diourbel (Oraj = 0.03 [0.02–0,04]), Louga (Oraj = 0,11 [0,06 − 0,19]), Fatick (Oraj = 0,27 [0,16 − 0,44]), Kaffrine (Oraj = 0,22 [0,15 − 0,32]) (see Table 3). Living in the North, South and South-East regions is a risk factor. (see Table 3))

The higher the socio-economic level, the more women are protected from FGM (Table 3).

**Table 3:** Factors associated with the development of FGM in Senegalese women

	FGM		
Characteristics	AOR	95% CI	*p*-value
**Year**	0,93	0,90 − 0,98	0,002
**Region**			
Dakar	1	-	
Ziguinchor	7,52	5,68 − 9,96	< 0,001
Diourbel	0,03	0,02 − 0,04	< 0,001
Saint-louis	1,56	1,06 − 2,29	0,023
Tambacounda	5,27	3,78 − 7,34	< 0,001
Kaolack	0,3	0,21 − 0,42	< 0,001
Thiès	0,21	0,15 − 0,28	< 0,001
Louga	0,11	0,06 − 0,19	< 0,001
Fatick	0,27	0,16 − 0,44	< 0,001
Kolda	7,58	5,43 − 10,6	< 0,001
Matam	11,7	8,96 − 15,2	< 0,001
Kaffrine	0,22	0,15 − 0,32	< 0,001
Kedougou	14,3	11,0–18,7	< 0,001
**Wealth index**			
Poorest	1	-	
Poorer	0,72	0,61 − 0,85	< 0,001
Middle	0,56	0,46 − 0,69	< 0,001
Richer	0,46	0,36 − 0,57	< 0,001
Richest	0,33	0,26 − 0,43	< 0,001
**Religion**			
Muslim	1	-	
Other	0,1	0,07 − 0,13	< 0,001

#### Daughters of women who have undergone female genital mutilation in Senegal between 2015 and 2019

The factors associated with the development of FGM in women’s daughters are the region of residence, the mother’s age, whether the mother has been circumcised, the mother’s level of education, the father’s level of education, the mother’s marital status, the mother’s religion and the socio-economic level of the household.

The risk factors were that the mother was circumcised (ORaj = 109 [82.4–145]), that the mother was married (ORaj = 1.36 [1.01–1.83]), the mother’s age and certain regions. These were the South, South-East and North regions (see Table 4).

The protective factors linked to the development of FGM among girls are the mother’s level of education and that of the mother’s husband/partner. The more educated the mother, the less likely the daughter is to be circumcised (primary level (ORaj = 0.75 [0.67–0.84]), secondary level (ORaj = 0.53 [0.44–0.63]) and university level (ORaj = 0.09 [0.03–0.24]). The higher the socio-economic level, the lower the risk of the woman’s daughter being circumcised. Another protective factor against circumcision is non-Muslim religion (ORaj = 0.5 [0.38–0.67] (see Table 4).

**Table 4:** Factors associated with the development of FGM in Senegalese girls

	FGM		
Characteristics	AOR	95% CI	*p*-value
**Year**	0,99	0,95 − 1,02	0,467
**Region**			
Dakar	1	-	
Ziguinchor	2,59	1,87 − 3,61	< 0,001
Diourbel	0,38	0,18 − 0,83	0,015
Saint-louis	3,17	2,24 − 4,49	< 0,001
Tambacounda	1,82	1,29 − 2,55	< 0,001
Kaolack	0,81	0,51 − 1,29	0,367
Thiès	0,66	0,42 − 1,03	0,069
Louga	0,95	0,58 − 1,54	0,828
Fatick	0,77	0,33 − 1,81	0,552
Kolda	2,13	1,49 − 3,04	< 0,001
Matam	3,91	2,87 − 5,32	< 0,001
Kaffrine	0,81	0,54 − 1,19	0,28
Kedougou	1,58	1,10 − 2,27	0,014
Sedhiou	2,57	1,82 − 3,63	< 0,001
**Age in 5-yea** **r groups**			
15–19	1	-	
20–24	4,14	3,24 − 5,29	< 0,001
25–29	9,09	7,19 − 11,5	< 0,001
30–34	14,3	11,2–18,2	< 0,001
35–39	14,2	11,1–18,1	< 0,001
40–44	12,3	9,43 − 15,9	< 0,001
45–49	9,19	7,14 − 11,8	< 0,001
**Highest educational level**			
No education	1	-	
Primary	0,75	0,67 − 0,84	< 0,001
Secondary	0,53	0,44 − 0,63	< 0,001
Higher	0,09	0,03 − 0,24	< 0,001
**Husband/partner’s education level**			
No education	1	-	
Primary	0,69	0,60 − 0,80	< 0,001
Secondary	0,62	0,49 − 0,79	< 0,001
Higher	0,83	0,54 − 1,26	0,377
**Respondent’s occupation (grouped)**			
Not working	1	-	
Working	1,06	0,97 − 1,16	0,193
**Wealth index**			
Poorest	1	-	
Poorer	0,77	0,69 − 0,86	< 0,001
Middle	0,55	0,48 − 0,63	< 0,001
Richer	0,39	0,32 − 0,49	< 0,001
Richest	0,3	0,22 − 0,42	< 0,001
**Current marital** **status**			
Not married	1	-	
Married	1,36	1,01–1,83	0,043
**Religio** **n**			
Muslim	1	-	
Other	0,5	0,38 − 0,67	< 0,001
**Respondent** **circumcised**			
No	1	-	
Yes	109	82,4–145	< 0,001

## Discussion

### FGM practice in Senegal from 2015 to 2023

Female genital mutilation is a harmful cultural practice [17]. Prevalence remains high in Africa. Our study showed that the prevalence of female genital mutilation in Senegal was 24.2% in 2015, 22.7% in 2016, 24.0% in 2017, 23.3% in 2018, 25.2% in 2019 and 20.1% in 2023. Although these prevalence rates are among the lowest in West Africa, with an average of 40%, they are also among the highest in the world [18] remain worrying because they are stagnating despite the many interventions carried out in Senegal. These interventions have followed one another since the promulgation of the law condemning FGM in 1999, with cases of repression and imprisonment [19]. As a result of these repressive measures, FGM has become a hidden practice [20]. Moreover, our study showed that the majority of women who are circumcised do so between the ages of 0 and 9, during infancy. Other studies have also shown that FGM is increasingly carried out at younger ages [21].

If mothers are circumcised, so are their daughters. Our study has shown that the prevalence among daughters of women who have been circumcised varies from 7.8% in 2015, 2016 (7.1%), 2017 (6.8%), 2018 (6.4%), 2019 (7.8%) to 6% in 2023. In comparison with other countries, prevalence rates are much lower in other sub-Saharan African countries, where the average is around 22.9% [22].

FGM is responsible for mutilation of the genital tract and serious complications. Several types of female genital mutilation have been found. In our study, the most frequent form of mutilation is “flesh removed from genital area”, the frequency of which is increasing, rising from 58.3% in 2015 to 76.5% of cases of mutilation in 2023. Infibulation (genital area sewn closed), which was the least practised at 7% in 2015, is also increasing, rising to 25.6% in 2023. However, infibulation, which is the narrowing of the vaginal orifice with the creation of a covering joint by cutting and apposition of the labia minora and/or the labia majora, with or without removal of the clitoris, is one of the most severe and serious forms of mutilation. Disinfibulation is often necessary to improve health and well-being and to allow sexual intercourse or facilitate childbirth [23]. Beyond the physical health complications of FGM that we have outlined, the psychological and social impacts are equally concerning. Studies by Mulongo et al. and Knipscheer et al. have documented significant psychological consequences [24, 25] including PTSD, depression, anxiety, and psychosexual problems among women who have undergone FGM. The social implications are complex and multifaceted – while FGM is often performed to increase marriageability and social acceptance within practicing communities, it can also lead to social isolation for women who suffer complications or for those who refuse the practice for their daughters in communities where it remains prevalent [26]. Understanding these psychosocial dimensions is crucial for developing holistic intervention approaches that address not only the physical aspects but also the mental health needs and social dynamics surrounding FGM.

In terms of practice, our study showed that most excisers are “traditional circumcisers”. In 2023, however, a new category of circumcisers emerged, which according to the respondents were health professionals (58, or 1.7%). This situation has been observed in several countries where FGM is traditionally practised and where the prevalence of medicalisation is increasing [27].

### Factors associated with changes in FGM between 2015 and 2023 among Senegalese women and their daughters in Senegal

Among the factors associated with the development of FGM among Senegalese women, our study found the region of residence. Thus, living in one region or another may be a protective factor or a risk factor. Living in a central region is a protective factor against the occurrence of FGM. These are the regions of Thiès (AOR = 0.21 [0.15–0.28]), Kaolack (AOR = 0.3 [0.21–0.42]), Diourbel (AOR = 0.03 [0.02–0.04]), Louga (AOR = 0.11 [0.06–0.19]), Fatick (AOR = 0.27 [0.16–0.44]), Kaffrine (AOR = 0.22 [0.15–0.32]). Living in the North, South and South-East regions is a risk factor. The same FGM hotspot regions had been identified by similar studies carried out before 2010 in Senegal [28]. As FGM is a highly cultural practice, the differences could be linked to the different cultural groups and their distribution in the regions of Senegal. In the central regions to the west, the majority of the population is of Wolof ethnicity [29] who do not practise FGM. In addition, living in the northern (Matam, Saint-louis), southern and south-eastern regions of the country, where the Poulars, Soninké and Diola ethnic groups are found in our study, is a risk factor. Other studies have shown similar results in Senegal, identifying the same regions at risk of FGM [30].

Another risk factor is socio-economic status. The higher a woman’s socio-economic status, the more protected she is from female genital mutilation. Other studies have shown that a high socio-economic level is a protective factor against FGM [31]. This study of several African countries also identified other socio-economic factors as protective against FGM, particularly for the daughters of circumcised mothers. These were the mother’s and father’s level of education.

The link between mother and daughter in terms of FGM is strong. Our study showed that the fact that the mother was circumcised predisposed the daughter to circumcision (AOR = 109 [82.4–145]). Other studies had already shown this relationship. Mothers are often faced with specific challenges related to the pressure exerted by the community and the family for their daughters to undergo FGM [32].

However, our study identified the mother’s level of education and that of the mother’s husband/partner as protective factors for daughters of circumcised mothers. The more educated the mother, the less likely the daughter was to be circumcised (primary level (AOR = 0.75 [0.67–0.84]), secondary level (AOR = 0.53 [0.44–0.63]) and university level (AOR = 0.09 [0.03–0.24]). A low level of maternal education increased the probability of FGM, while a high level of education on the part of the father protected the daughter from FGM [33]. This finding aligns with several studies. Hayford et al. demonstrated that educated parents are more likely to question traditional practices and have greater exposure to anti-FGM messaging [34]. Education empowers parents with critical thinking skills and often reduces the influence of traditional authority figures who perpetuate FGM [35]. Additionally, educated parents typically have wider social networks beyond traditional communities, exposing them to diverse perspectives on child-rearing practices [36].

### Limitations

This study has several limitations that must be acknowledged. First, the analysis relies on cross-sectional survey data, which limits the ability to establish causal relationships between the explanatory variables and the practice of FGM. The observed associations should therefore be interpreted with caution.

Second, all information related to FGM—both for women and their daughters—was self-reported, which introduces a risk of recall bias and social desirability bias. Given the criminalization and increasing stigmatization of FGM in Senegal, some respondents may have underreported their own or their daughters’ circumcision status.

Third, the type of FGM practiced was reported by the women themselves, without any medical verification. This may result in misclassification, especially in distinguishing between forms of cutting or understanding the medicalization of the practice.

Finally, this study only examines quantitative determinants of FGM. A qualitative component could help explore the complex sociocultural and intergenerational norms that drive the persistence of FGM despite legal bans and public health interventions.

Despite these limitations, the study’s use of nationally representative DHS data enhances the generalizability of the findings to Senegalese women of reproductive age and their daughters.

## Conclusion

In Senegal, the evolution of FGM from 2015 to 2023 appears to be stagnant, with prevalence levels showing little variation and remaining concentrated in the same hotspot regions. The findings suggest that maternal circumcision is strongly associated with an increased likelihood of daughters also undergoing FGM. Conversely, higher levels of household wealth and parental education appear to be associated with a lower risk of circumcision in girls.

These results may inform future interventions. Policymakers could consider implementing regionally tailored strategies that address the socio-cultural and economic determinants of FGM. Such strategies may include community-based awareness initiatives, improved capacity of health and child protection services, and sustained educational efforts. Previous research has highlighted the potential value of multisectoral collaborations involving health, education, legal, and social sectors, as well as engagement with community and religious leaders.

Finally, future studies—particularly those employing qualitative or mixed-methods approaches—could help uncover the sociocultural dynamics that remain hidden in quantitative surveys and provide deeper insights into the persistence of FGM in Senegal.

## Data Availability

The data supporting the findings of this study are publicly available and can be accessed through the DHS Program at the following link: https://dhsprogram.com/data/available-datasets.cfm.

## Abbreviations

CI: Confidence intervals
CNERS: Senegal National Ethics Committee of Research
DHS: Demographic Health Survey
FGM: Female genital mutilation
IR: Individual record
ORaj: Adjusted Odds ratio
SE: Standard error
